# Evaluating the impact of leniolisib treatment on symptoms and health-related quality of life in activated phosphoinositide 3-kinase delta (PI3Kδ) syndrome

**DOI:** 10.3389/fimmu.2026.1739437

**Published:** 2026-05-07

**Authors:** V. Koneti Rao, Allison Morgan, Troy R. Torgerson, Amanda Harrington, John Whalen, Ewen Munro, Jo Luscombe, Anna de la Motte, Beverly Romero, Roxana Bahar, Jason Bradt

**Affiliations:** 1 Laboratory of Clinical Immunology and Microbiology, National Institutes of Health, Bethesda, MD, United States; 2 Metis Clinical Ltd., Nottingham, United Kingdom; 3 Allen Institute for Immunology, Seattle, WA, United States; 4 Pharming Healthcare Inc., Warren, NJ, United States; 5 Pharming Group N.V., Leiden, Netherlands; 6 Sprout Health Solutions Ltd., Los Angeles, CA, United States

**Keywords:** APDS, burden of disease, HRQOL, leniolisib, PASLI, PI3Kδ inhibitor

## Abstract

**Purpose:**

Activated phosphoinositide 3-kinase delta (PI3Kδ) syndrome (APDS) is an ultra-rare inborn error of immunity, characterised by immune deficiency and dysregulation. In a randomised controlled trial (RCT; NCT02435173) and open-label extension (OLE; NCT02859727), leniolisib, a selective PI3Kδ inhibitor, was efficacious and well-tolerated in individuals with APDS. These trials suggested some improvements with leniolisib in health-related quality of life (HRQoL) using generic instruments, with other anecdotal evidence also describing improvements in specific domains. Here, all available qualitative data relating to treatment experience were systematically assessed to evaluate any perceived impacts of leniolisib on patients’ lives.

**Methods:**

Changes in APDS-related symptoms and patient HRQoL were assessed using unsolicited qualitative data captured from 36 leniolisib-treated individuals: RCT and OLE clinician-recorded, open-text patient narratives (n=31); case reports (n=4); standalone qualitative study conducted with APDS patient/caregiver interviews/narratives (n=1).

**Results:**

Improvements in APDS-related symptoms and HRQoL were reported following treatment with leniolisib: 86.1% (31/36) of individuals mentioned improvements in ≥1 symptom/HRQoL impact. Of these, 87.1% (27/31) explicitly attributed ≥1 improvement to leniolisib.

One-third (12/36) of individuals explicitly attributed improvements in fatigue/energy to leniolisib. This was associated with reported HRQoL improvements explicitly attributed to leniolisib: physical activity (33.3% [12/36]), work/school performance/attendance (13.9% [5/36]) and travel ability (8.3% [3/36]). Moreover, 36.1% (13/36) of participants explicitly attributed improvements in their overall wellbeing to leniolisib.

**Conclusion:**

As data were unsolicited and baseline symptoms/HRQoL were not available, results may not represent all changes experienced, and is not possible to determine the amount of change in symptoms/HRQoL associated with leniolisib. Nevertheless, these analyses indicate that participants with APDS experienced improvements in symptoms and overall wellbeing following treatment with leniolisib.

## 1 Introduction

Activated phosphoinositide 3-kinase delta (PI3Kδ) syndrome (APDS) is an ultra-rare inborn error of immunity characterised by immune deficiency and immune dysregulation (1–3). APDS is a recently characterised disease with the causative genes for APDS1 and APDS2 only being identified in 2013 and 2014, respectively, and only 351 reported cases in the literature (4, 5). It is a progressive disease caused by autosomal dominant mutations in the genes encoding the PI3Kδ subunits, resulting in hyperactive signalling within this pathway (1, 6, 7). PI3Kδ is central in regulating a wide range of fundamental cellular processes (7). Patients, therefore, experience a wide variety of clinical manifestations with varying severities. These include recurrent sinopulmonary infections, persistent viral infections, autoimmune disorders and non-neoplastic lymphoproliferation, which are associated with a range of symptoms including cough, congestion, muscle aches, and fever (1, 6, 8). Many patients can progress to severe gastrointestinal (GI) symptoms, impaired lung function, and lymphoma (1, 6, 8). Overall, the variety of clinical manifestations and symptoms can result in a heterogenous lived experience of patients with APDS, with broad impacts on an individual’s health-related quality of life (HRQoL) (1, 6, 8–11).

For individuals with APDS, declining HRQoL can begin in childhood and continue throughout adulthood, affecting work/educational, physical, social, mental and emotional domains (6, 9–13). As a consequence of persistent symptoms, individuals with APDS can experience extreme fatigue, requiring lifestyle adjustments, as they are unable to exercise or complete everyday tasks (10). Depending on the severity of symptoms, individuals may also have to undergo periods of isolation, practice careful hygiene and avoid contact with animals and with people who may have infections, due to their weakened immune system (9, 10, 13–15).

The progressive nature of APDS means that individuals can experience a rapid accumulation of clinical manifestations, some of which may lead to irreversible end-organ damage (3, 16–18). Accurate, timely diagnosis of APDS is also challenging, reflected by a mean diagnostic delay of 10.6 years (5). Due to the multi-system manifestations and the sporadic nature and variability of symptom onset, individuals may be referred to and lost among several different specialists receiving various symptomatic, empirical treatments whilst pursuing an accurate diagnosis (7, 16, 19). Together, these factors can perpetuate the negative impact on HRQoL, with patients experiencing reduced HRQoL for longer periods (3, 16, 17).

A range of treatments are currently used for APDS, with most primarily alleviating symptoms associated with either immune deficiency (such as antibiotics and immunoglobulin replacement therapy) or immune dysregulation (such as immunosuppressants and surgeries) (6, 7, 20, 21). Haematopoietic stem cell transplantation (HSCT) can be a potential cure for some individuals; however, it is not the preferred treatment for the majority of affected individuals because of the substantial morbidity and mortality risks, coupled with limited donor availability and patient comorbidities that may preclude transplantation as an option (7, 22–24). Therefore, there is a clear need for effective, licensed therapies addressing the underlying cause of the disease.

Leniolisib is an oral, selective PI3Kδ inhibitor that targets the underlying pathophysiology of APDS (25). In a randomised, placebo-controlled trial (RCT; NCT02435173, Study 2201) and its open-label extension (OLE; NCT02859727, Study 2201E1), leniolisib was efficacious and well-tolerated, significantly reducing lymphoproliferation and improving immunophenotypes compared with placebo (25, 26). Subsequently, leniolisib has been approved in the United States, the United Kingdom (UK), Israel and Australia as a treatment for APDS in individuals ≥12 years of age (25, 27–30).

There are currently no disease-specific HRQoL tools to capture patient-reported outcomes for APDS (31, 32). Therefore, the RCT used generic HRQoL measurement tools: 36-item Short Form Health Survey (SF-36) and Work Productivity and Activity Impairment plus Classroom Impairment Questionnaire (WPAI+CIQ). At Day 85, there were no statistically significant differences between the leniolisib and placebo groups in patient- and clinician-reported outcomes. However, clinically meaningful improvements were seen in the leniolisib arm for both patient- and physician-general assessments, while the placebo arm improved meaningfully only for the physician-general score. Furthermore, the WPAI-CIQ demonstrated clinically meaningful improvements for the leniolisib arm but not the placebo arm (25). These results align with physician reports of various positive HRQoL impacts of leniolisib (33). This suggests that the generic HRQoL measurement tools may be too insensitive to detect objective and subjective changes in APDS. This has been a consistent challenge within rare diseases, with growing emphasis, supported by the FDA and European Medicines Agency, on developing new and innovative methods to collect and assess HRQoL data to ensure that the patient experience is reported and patient voices are heard (34–37).

Within this study, qualitative patient data were collected and analysed from individuals with APDS being treated with leniolisib, as a new method to assess HRQoL and to evaluate leniolisib’s impact on the daily lives of patients. Results of the qualitative patient data analyses were used in conjunction with a targeted literature review (TLR), to develop a conceptual framework of APDS symptoms, manifestations and HRQoL impacts.

## 2 Methods

### 2.1 Targeted literature review

A TLR was performed to understand the burden of disease associated with APDS as reported in the literature. The TLR was conducted via PubMed on the 8^th^ of September 2023. Search terms used included ‘activated phosphoinositide 3-kinase delta syndrome’ OR ‘APDS1’ OR ‘APDS2’ OR ‘activated phosphoinositide 3-kinase-δ syndrome’ OR ‘activated PI3K-delta syndrome’, with the addition of the terms ‘qualitative’ OR ‘focus group*’ OR ‘interview*’ OR ‘patient experience*’. Articles returned through the searches were reviewed for data regarding clinical manifestations of APDS, symptoms and HRQoL impacts, and were excluded if they did not contain information on the patient experience and symptoms/impacts of APDS. The findings from the TLR were subsequently used to inform the conceptual model.

### 2.2 Qualitative patient data

In order to identify all available qualitative patient data that spontaneously reports leniolisib treatment experiences, the following sources were collated: clinician-reported, open-text, unstructured narratives collected during the leniolisib clinical trials (RCT [Part 1 (dose finding trial; DFT) and Part 2] and OLE Study), four case reports, and a standalone qualitative interview study conducted with patients with APDS and their caregivers in 2023 (25, 26, 33).

During the leniolisib RCT and OLE, the unstructured narratives were completed by clinicians at the end of Part I (12-week treatment period of leniolisib 10 mg twice a day [BID] from Day 1–Day 28, then 30 mg BID from Day 29–Day 56, and then 70 mg BID from Day 57–Day 84) and Part II (12-week treatment period of leniolisib 70 mg BID from Day 1–Day 85) of the leniolisib RCT and during patient visits in the OLE (data from up to November 2022 were included for patients receiving leniolisib 70 mg BID), based on their assessments and discussions with patients (25). No narratives were collected at baseline. To be included in the current analysis of treatment experience, the narratives were required to represent patients who had received leniolisib, and the clinical notes had to be legible.

In addition to the unstructured narratives, data were also collected from four case reports of individuals with APDS receiving leniolisib included three patients who received leniolisib through pre-license individual patient access (one on leniolisib 60 mg/day for approximately 1.5 years, one on 70 mg BID for approximately 6.0 months, and one patient also in an individualised curative trial who received leniolisib 70 mg BID for approximately 1.5 years) and one patient from Part 1 of the RCT and the OLE (for whom a narrative was not available from the RCT and OLE and has therefore been included as a case report) (25, 38–41).

The standalone qualitative interview study was conducted with healthcare providers, individuals with APDS and caregivers to investigate the symptoms and HRQoL impacts of living with APDS. Study methodology has been previously published by Hitchcock et al. (33).

### 2.3 Analysis of qualitative patient data

Narratives from the clinical trials, case reports and standalone qualitative interview study on leniolisib treatment experiences, were extracted, reviewed and every independent statement within each narrative was assigned a qualitative content code that reflected the substance of that statement. The coding and analysis followed principles in line with qualitative thematic (content) analysis (42), with additional features drawn from grounded theory (43), conforming to the guidelines for best practice in qualitative research (44).

Data were imported and analysed in a qualitative analysis software (MAXQDA™ 2022 [VERBI Software]) (45). The first round of analysis involved the development of a coding template to capture any potentially relevant disease concepts (e.g. symptoms, impacts, clinical manifestations, and overall burden of APDS) and treatment-related concepts (e.g. adverse events while on leniolisib, improvement or worsening explicitly or implicitly attributed to leniolisib treatment, changes in symptoms or impacts since baseline) that emerged from the data. When classifying the impact of leniolisib on symptoms or HRQoL, explicit attribution was defined by direct mention of leniolisib, study drug, or change from baseline: for example, *“When asked what has changed in his life since he started taking the investigational medicinal product [IMP; leniolisib], he notes, ‘my energy is higher’…”.* Implicit attribution was defined by a description of changes that occurred after starting treatment but were not explicitly attributed to leniolisib or the time since starting treatment: for example, *“Her mood has also improved prompting her local physician to discontinue her anti-depressant.”*.

**Figure 1:**
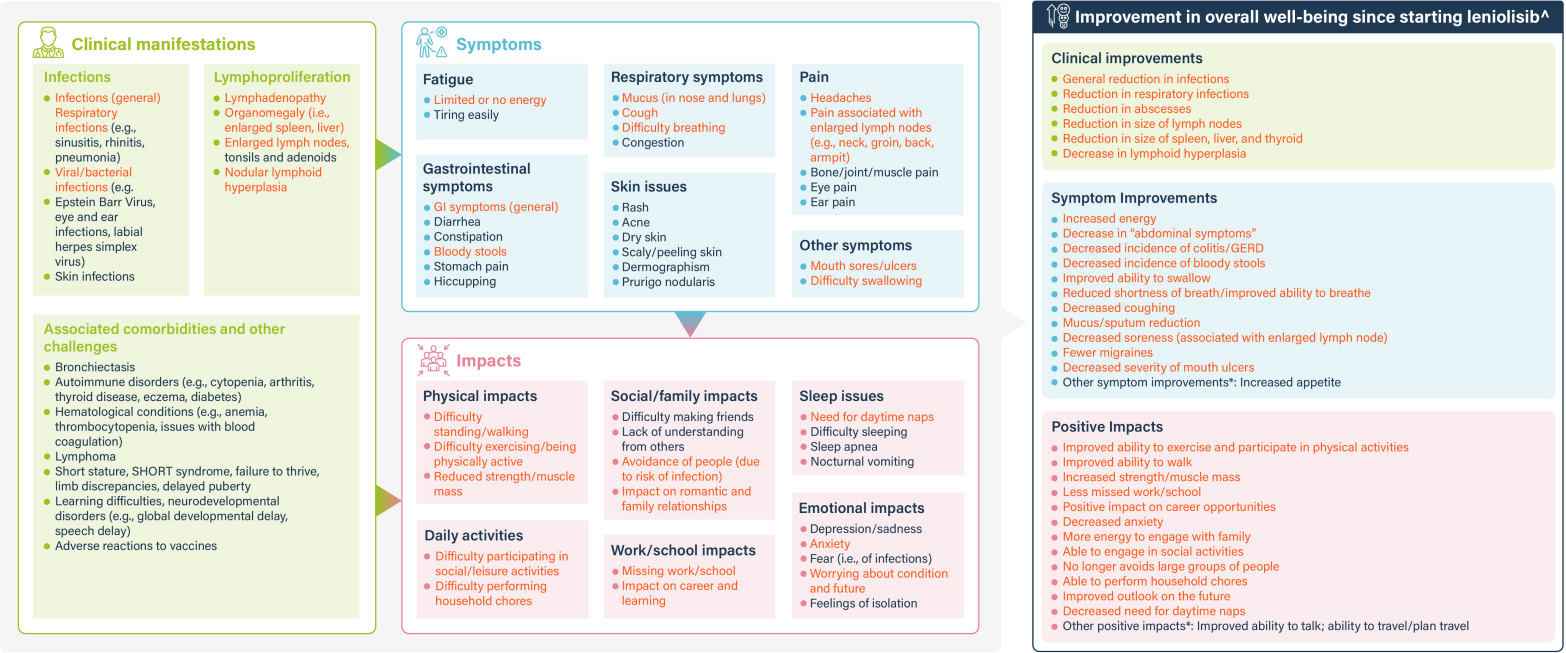
Conceptual model of clinical manifestation, symptoms, and impacts of APDS. Orange text represents clinical manifestations and concepts associated with APDS that were described as improving during treatment with leniolisib. ^Includes improvements in clinical manifestations and APDS related symptoms and impacts explicitly discussed in relation to leniolisib or the treatment period. *Symptom improvements/positive impacts related to concepts not identified in the literature or qualitative patient/caregiver interviews. APDS, activated phosphoinositide 3-kinase delta syndrome; GERD, gastroesophageal reflux disease; SHORT, short stature, hyperextensibility of joints, ocular depression, Rieger anomaly, and teething delay.

Coding discrepancies were reconciled by modification or re-definition of the coding template. The second round of analysis identified key themes, attributes and relationships (as shown in **Figure 1**) into which concepts from the RCT/OLE unstructured narratives, case studies and standalone qualitative interviews were grouped. The analysis of source documents was overseen by an independent reviewer. All final counts were reviewed independently by two individuals to ensure accuracy and consistency.

### 2.4 Development of conceptual model

To construct an understanding of the impact of APDS on individuals, symptoms, clinical manifestations and HRQoL impacts were included in a conceptual model which was developed using the qualitative patient data analysis [leniolisib clinical trials, case reports and qualitative interview study (33)] and the TLR.

## 3 Results

### 3.1 Targeted literature review

The TLR returned 112 articles, 106 of which were excluded because they did not contain information on the patient experience and symptoms/impacts of APDS. Six articles were selected for data extraction: one systematic review (20), two evidence reviews (3, 46), one case study review (47), and two cohort studies (48, 49). Where no patient-reported information on burden of disease was available, data on the clinical manifestations (e.g. infections, lymphoproliferation and comorbidities) of APDS were extracted as proxy measures of the burden of illness.

### 3.2 Qualitative patient data

Overall, qualitative patient data were available for 36 leniolisib-treated individuals: 31 participants from the leniolisib RCT and OLE trials, four participants from the case reports, and one from the standalone qualitative study. One individual participated in both the standalone qualitative study and the RCT and OLE (but has only been counted in the sample size for the RCT and OLE trials [n=31]). For this individual, data from the patient interview and caregiver narrative collected in the standalone qualitative study were combined with the narrative from the RCT, and the quotes analysed together.

From the RCT and OLE, all 37 trial participants had clinician-reported open-text narratives available, representing data collected during clinical visits between December 2015 and December 2022. Of these 37 participants, two were excluded from the analysis due to narratives only being available for placebo visits, and four were excluded due to handwriting being illegible. Therefore, 31 participants were included from the RCT and OLE, with 19 of these participants having data available from both studies.

Of the four case reports, three reported on individuals who received leniolisib through pre-license individual patient access (one individual being a post-haematopoietic stem cell transplantation case report) and one reported on an individual from the DFT (Part 1 of the RCT) and OLE (25, 38–41).

From the standalone qualitative study conducted with healthcare providers, patients with APDS and caregivers, qualitative interviews and written narratives, two patients who had received leniolisib were included (33). One individual was represented by just a patient interview, and the other was represented by a patient interview, caregiver narrative and a narrative available from the RCT (and is included in the count of 31 patients from the RCT).

### 3.3 Conceptual model of clinical manifestations, symptoms and impacts of APDS

Key clinical manifestations, symptoms, and HRQoL impacts, reflecting the burden of disease for APDS, were identified from both the TLR and qualitative patient data, grouped for analysis, and used to develop the conceptual model presented in **Figure 1**. Common clinical manifestations of APDS included frequent infections and lymphoproliferation, which led to multi-system symptoms and related impacts on HRQoL. Commonly reported symptoms resulting from the clinical manifestations of APDS included fatigue, body pain, gastrointestinal symptoms, respiratory problems, difficulty swallowing, skin disorders, and infections. HRQoL impacts included challenges in completing activities of daily living (such as walking, standing and household chores), significant work and school impacts, sleep disturbances, family and social impacts, alongside emotional impacts.

### 3.4 Treatment experience

Overall, leniolisib was associated with a positive impact on APDS symptoms and HRQoL, with 31/36 (86.1%) participants spontaneously reporting improvement in ≥1 symptom or a positive HRQoL impact. Of these, 27/31 (87.1%) participants explicitly mentioned attributing ≥1 of their improvements to leniolisib.

#### 3.4.1 Symptom experience

Patient narrative quotes reporting symptom improvements are presented in **Supplementary Materials 1**. In terms of symptom experience reported, 21/36 participants (58.3%) had ≥1 symptom-related improvement with explicit or implied attribution to leniolisib (**Table 1**). For these 21 participants, the mean noted number of improved symptoms per person was 1.9 (range 1–5). Among the 36 reports, 13/36 (36.1%) did not discuss changes in any symptoms and 2/36 (5.6%) mentioned only experiencing worsening of symptoms. Amongst only the individuals with narratives from the DFT/RCT and OLE (30/36 [83.3%]), 18/30 (60.0%) had ≥1 symptom-related improvement with explicit or implied attribution to leniolisib (**Supplementary Material 2**).

**Table 1:** Individual patient overview of symptom improvements or worsening while on leniolisib.

Patient number	Fatigue	GI symptoms	Swallowing	Breathing	Appetite	Pain/ soreness	Migraine	Mouth ulcers	Coughing/ sputum
1									
2									
3									
4									
5									
6									
7									
8									
9									
10									
11									
12									
13									
14									
15									
16									
17									
18									
19									
20									
21									
22									
23									
24									
25									
26									
27									
28									
29									
30									
31									
32									
33									
34									
35	*								
36									
	Improvement explicitly attributed to leniolisib								
	Improvement with implied attribution to leniolisib								
	Patients did not discuss any symptoms								
	Worsening with implied attribution to leniolisib								
	Worsening explicitly attributed to leniolisib								
	Specific symptom was not mentioned								

*Initial worsening of fatigue (in the first two months) that resolved in the third month of treatment. GI, gastrointestinal.

**Figure 2:**
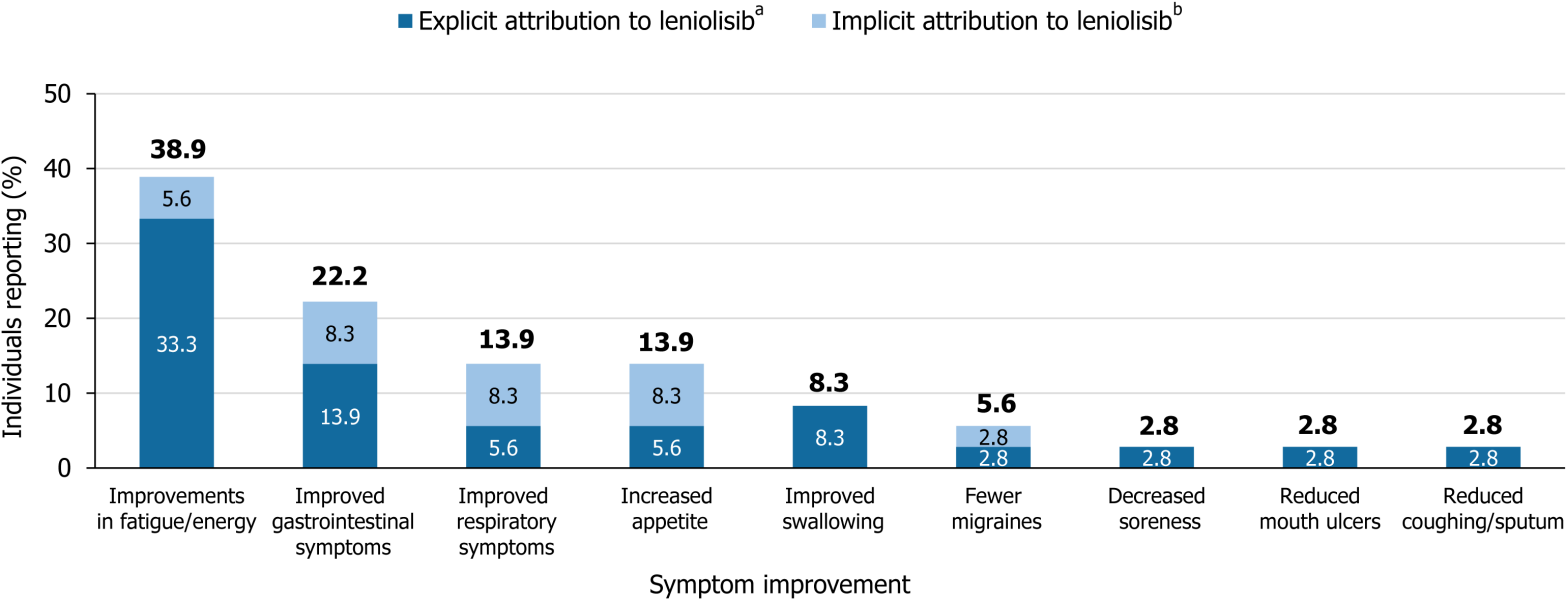
Reported symptom improvement with explicit or implied attribution to leniolisib. Data from clinical trials, case reports, and qualitative interviews with patients with APDS, caregivers, and clinicians (N = 36). The proportions of patients affected by each symptom at baseline were not available. ^a^Explicit attribution was defined by direct mention of leniolisib, study drug, or change from baseline. ^b^Implicit attribution was defined by a description of changes that were not specifically related to leniolisib or the time since starting treatment. APDS, activated phosphoinositide 3-kinase delta syndrome.

Improvements in fatigue/energy were spontaneously reported for 14/36 (38.9%) of the participants for whom qualitative data were available, with 12/36 (33.3%) explicitly attributing improvements in fatigue/energy to leniolisib (**Figure 2**). Other noted symptom improvements included improved GI symptoms (8/36, 22.2%), improved respiratory symptoms (5/36, 13.9%) and increased appetite (5/36, 13.9%), with at least half of these improvements being explicitly mentioned as attributed to leniolisib. Furthermore, 3/36 (8.3%) of patients stated reduced problems with swallowing, all of which were noted to be explicitly attributed to leniolisib. Similar numerical patterns were observed when considering the participants with narratives from the DFT/RCT and OLE (n=30), presented in **Supplementary Material 2**.

#### 3.4.2 HRQoL impacts

Improved functioning across multiple areas of daily life was noted in several participant narratives. The descriptions ranged from general notes regarding activity levels to very specific examples of how the patient’s activity and daily functioning had improved. Patient narrative quotes of HRQoL improvements from these patients are presented in **Supplementary Materials 3**.

Analysis of unsolicited reports of improvement or worsening of HRQoL showed that 27/36 (75.0%) participants mentioned ≥1 HRQoL improvement explicitly or implicitly attributed to treatment with leniolisib (**Table 2**). Among these 27 participants, the mean number of improvements experienced per participant was 2.3 (range 1–5); with one participant mentioning worsening of a HRQoL domain being attributed to leniolisib (emotional impacts). For 9/36 participants change in any HRQoL factor was not discussed. Amongst only the individuals with narratives from the DFT/RCT and OLE, 23/30 (76.7%) had ≥1 HRQoL improvement with explicit or implied attribution to leniolisib.

**Table 2:** Individual patient overview of HRQoL improvements or worsening while on leniolisib.

Patient number	General well-being	Physical impacts	Work/social impacts	Emotional impacts	Travel	Family/social impacts	Household chores	Need for daytime sleep	Ability to talk
1									
2									
3									
4									
5									
6									
7									
8									
9									
10									
11									
12									
13									
14									
15									
16									
17									
18									
19									
20									
21									
22									
23									
24									
25									
26									
27									
28									
29									
30									
31									
32									
33									
34									
35									
36									
	Improvement explicitly attributed to leniolisib								
	Improvement with implied attribution to leniolisib								
	Patients did not discuss any HRQoL ‘domains’								
	Worsening with implied attribution to leniolisib								
	Worsening explicitly attributed to leniolisib								
	Specific HRQoL ‘domain’ was not mentioned								

HRQoL, health-related quality of life.

**Figure 3:**
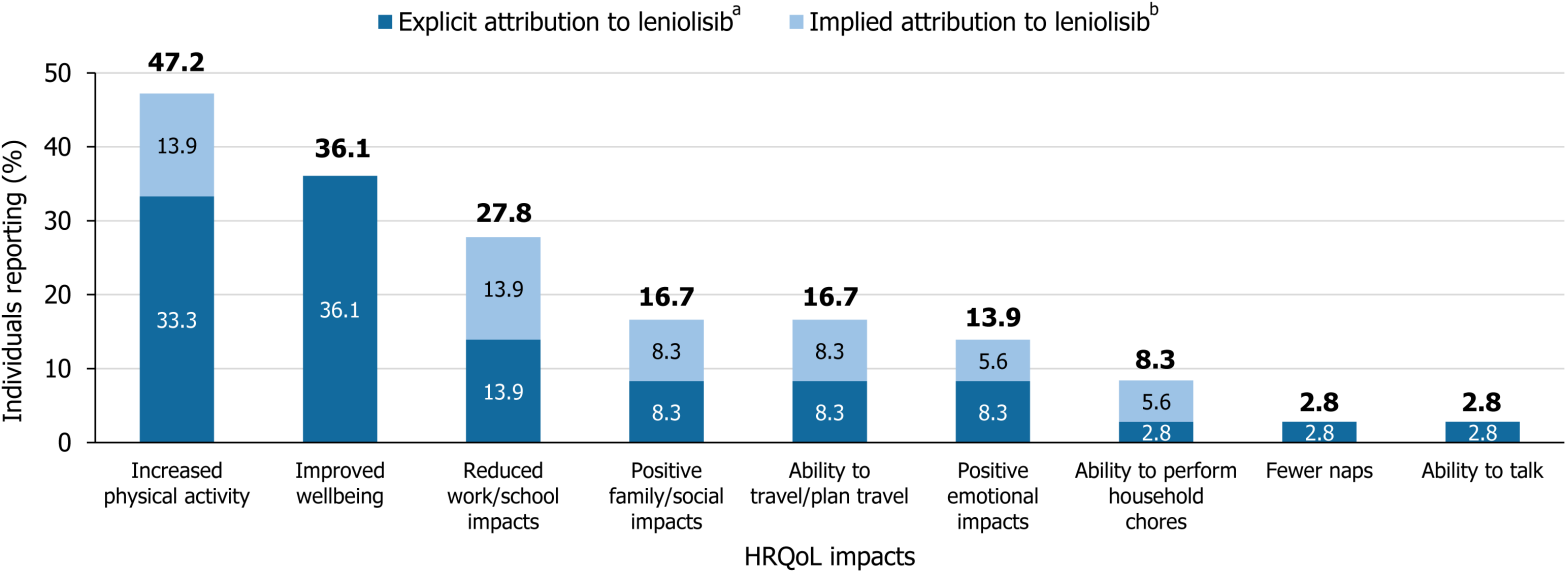
Reported positive HRQoL impacts with explicit or implied attribution to leniolisib. Data from clinical trials, case reports, and qualitative interviews with patients with APDS, caregivers, and clinicians (N = 36). The proportions of patients affected by each symptom at baseline were not available. ^a^Explicit attribution was defined by direct mention of leniolisib, study drug, or change from baseline. ^b^Implicit attribution was defined by a description of changes that were not specifically related to leniolisib or the time since starting treatment. APDS, activated phosphoinositide 3-kinase delta syndrome; HRQoL, health-related quality of life.

HRQoL improvements of increased physical activity were spontaneously reported for 17/36 (47.2%) participants, with 12/36 (33.3%) participants explicitly attributing improvements to leniolisib treatment (**Figure 3**). Moreover, 13/36 (36.1%) participants explicitly attributed improvements in overall wellbeing to leniolisib. Improvements in work or school performance or attendance were noted for 10/36 (27.8%) participants, and 6/36 (16.7%) patients spontaneously reported an improvement in ability to travel and plan travel. In both HRQoL domains, half of the participants experiencing an improvement explicitly attributed this to leniolisib. Similar numerical patterns were observed when considering the narratives of participants in the DFT/RCT and OLE (n=30), presented in **Supplementary Material 4**.

## 4 Discussion

This study highlights the importance of using different methods to collect and analyse HRQoL data, to understand the burden of APDS with a patient-centric view. While the RCT and OLE, which primarily assessed the clinical benefits of leniolisib, reported limited HRQoL outcomes and improvements using generic patient-reported outcome measures (SF-36 and WPAI+CIQ), the qualitative patient data analysed here provide further insight into how leniolisib, as early as 12 weeks after the initiation of treatment, may be associated with improvements in symptoms (e.g. reducing fatigue) and HRQoL (e.g. ability to go to work and school) for people with APDS. This demonstrates how novel methods like patient interviews from clinical studies can act as valuable alternatives for exploring the burden of disease and patients’ perceptions of treatment benefit, in the absence of large, quantitative studies which may be unfeasible for ultra-rare diseases (34–37, 50).

This study is one of the first to assess the HRQoL impacts of living with APDS, and to provide an insight into patients’ perspectives of key areas that affect their daily activities. The TLR revealed sparse data on the burden of APDS in the literature, highlighting the need for qualitative patient data to better understand APDS disease burden. The conceptual model, informed by the qualitative data analyses, and clinical manifestations identified from the TLR as proxies for patient experience (**Figure 1**), shows that the burden of disease in patients with APDS impacts many areas including HRQoL and activities of daily living. These findings align with a recent vignette-based study by Tutein Nolthenius et al., which showed that APDS has a substantial HRQoL impact (32). This is also supported by the conceptual model developed by Hitchcock et al., that used data from an interview study with healthcare providers, individuals with APDS and caregivers to model the experience of APDS, and a survey published by Immunodeficiency UK and the National Institute of Health and Care Excellence (NICE), that asked individuals with APDS, caregivers and family members to report the impact of APDS on their lives (13, 33). Understanding the impacts that APDS has on patients is crucial for developing and assessing targeted interventions that can improve the overall wellbeing and daily functioning of individuals living with APDS and other rare diseases.

The data collated here demonstrate a variety of real-world benefits to patients’ daily lives that were reported to be associated with leniolisib treatment, with improvements in symptoms and HRQoL across several domains including reducing fatigue and increasing exercise tolerance. It is important to note that with the absence of baseline data prior to leniolisib initiation, it was not possible to ascertain the proportion of patients affected by each symptom and HRQoL impact pre-treatment, and subsequently the magnitude of change after leniolisib use could not be measured. Nevertheless, these spontaneous reports offer valuable insights into qualitative improvements in symptoms and HRQoL impacts, attributed explicitly or implicitly to leniolisib use, providing a more in-depth understanding of APDS disease burden and patients’ treatment experiences.

One significant symptom of APDS is fatigue and decreased energy, which can be caused by autoimmune manifestations, and results in reduced patient activity. It is therefore promising that almost 40% of patients spontaneously reported improvements in fatigue/energy, with most of these patients explicitly attributing the improvement to leniolisib. Similarly, leniolisib treatment was associated with HRQoL improvements, including increased physical activity, reduced impacts on work and school, and an increased ability to travel/plan travel. These data are supported by results from a survey of physicians treating patients with APDS with leniolisib through pre-license individual patient access, which reported that 15/30 patients experienced chronic fatigue pre-leniolisib, with 12/15 (80%) of these patients experiencing ‘meaningful improvements or better’ with leniolisib treatment, and no patients experiencing ‘worsening or new’ chronic fatigue (38). Taken together, these data highlight the association between leniolisib treatment and improvement in fatigue, a restrictive and impactful symptom.

The RCT previously demonstrated that leniolisib was well-tolerated and an effective treatment in both adolescents (ages 12–17 years) and adults (ages ≥18) with APDS, reducing lymphoproliferation and improving immunophenotypes compared with placebo (51). This is consistent with the improvements in symptoms and HRQoL observed in the qualitative data presented here, which include individuals of at least 12 years of age, and one paediatric individual (who received leniolisib through pre-license individual patient access). Symptom and HRQoL improvements that occurred in younger individuals who were treated with leniolisib suggest that earlier treatment may be important in reducing accumulation of active symptoms and progressive disease throughout adulthood, potentially benefiting patient HRQoL in the longer term. Emerging data from case series of paediatric patients treated with leniolisib suggest that leniolisib may be associated with positive symptom and HRQoL impacts in individuals with APDS aged <12. A Russian study, including six leniolisib-treated paediatric (aged <12) and four adolescent (aged ≥12) patients, reported improvements after 3 months in lymphoproliferation (10/10 patients) and immune phenotype (increased naive B cells: 6/9 patients; normalisation of immunoglobulin M: 6/7 patients), reductions in annual infection rate and improvements in HRQoL (assessed using the Paediatric QoL inventory [PedsQL] after 6 months of therapy in 6/6 patients studied) (52). Other paediatric case studies, including one from Spain (two patients aged 11 when starting leniolisib) and one from Switzerland (one patient aged 9), reported increased activity levels and reduced fatigue, and improved QoL, respectively (53, 54). Further research is required to confirm the impact of both early and long-term treatment in younger individuals, with ongoing paediatric clinical trials currently investigating the safety and efficacy of leniolisib in children with APDS aged 1−6 (NCT05693129) and 4–11 years (NCT05438407) (55, 56).

### 4.1 Study limitations

Qualitative data were extracted from narratives collected through several study types, meaning there is likely heterogeneity amongst the included participants; as no baseline data were available for the qualitative analysis, it was not possible to demonstrate that participants were comparable between studies at baseline. Additionally, patient-level data were taken from four case studies, and inherent selection bias of case studies towards atypical cases may limit representativeness of the case study data (57). With strict eligibility criteria for enrolment into the RCT and OLE, it is expected that participants from this cohort were more homogenous (25, 26), and so assessing narratives from these studies alone may facilitate a clear view of the treatment effect, distinguished from baseline differences. Data showed similar percentages of individuals reporting symptom-related (60.0% vs 58.3%) and HRQoL improvements (76.7% vs 75.0%) in the group of patients from the RCT/OLE vs the total cohort of 36 patients for whom qualitative data were identified. This suggests that the heterogenous baseline across included studies does not have a large effect on the results of the study and even if data from the case studies were removed, observed patterns in improvements in symptoms and HRQoL attributed to leniolisib, remained.

Key limitations of the data also include that symptoms and HRQoL impacts were not objectively and systematically reported in the source materials. Only unprompted reports of improvements or worsening in symptoms and HRQoL, that were mentioned by participants, were collected, with 13/36 participants not discussing symptoms in the narratives. As patients were not specifically asked about certain symptoms or HRQoL impacts, this analysis does not reflect all the benefits that patients experienced from leniolisib treatment, as some changes may not be mentioned in the narratives. Furthermore, some participants only had narratives from the RCT, which were collected at Week 12, with any potential further improvements with longer-term treatment not being captured. Another limitation is that bias may have been introduced into the reporting of some of the patient data as clinicians and participants were not blinded to treatment within the OLE, the case reports, and the standalone study; in these cases perceived changes in symptoms or HRQoL may in part have been related to a placebo effect (26, 33). Finally, patient experiences captured in this study were only available from a small cohort, so may not be reflective of all individuals living with APDS globally; however, given the rare nature of this condition and general lack of natural history data, the size of the sample reflected here is representative and appropriate for the study’s objective.

With regards to study methodology, correlation analysis was performed to understand association (including explicit attribution) between leniolisib treatment and changes in symptoms and HRQoL; however, causality cannot be concluded. Future studies are required to enhance the understanding of the correlation between symptoms and HRQoL in APDS. Understanding patients’ treatment experiences alongside results from clinical studies can ensure that both the full therapeutic benefits and potential challenges experienced by patients are thoroughly evaluated, leading to a more holistic understanding of the impacts of leniolisib on patients’ lives.

## 5 Conclusion

Analyses of qualitative data on patient experiences from clinical trials and real-world settings shown here indicate that leniolisib treatment not only provides clinical benefits but is attributed to positive impacts on symptoms and overall HRQoL for patients with APDS. In turn, these positive impacts observed in leniolisib-treated patients may lead to an overall decrease in disease burden of APDS. This study highlights the importance of tailoring and leveraging practical and innovative approaches to assess disease-specific HRQoL impacts beyond those included in standardised assessments during clinical trials for rare diseases, to ensure that the patient experience can be adequately captured and reported, assuring that the patient voice is heard. **Supplementary Material 5** provides a plain English summary of why this study was needed, its results and what they mean.

## Data Availability

Pharming Group N.V. is committed to responsibly share data generated by interventional clinical trials with researchers. Researchers who have a methodologically sound research proposal should send inquiries or requests to info@pharming.com. Data requestors may be required to sign a data access agreement.
