# Supplementary material for: Evaluating the impact of leniolisib treatment on symptoms and health-related quality of life in activated phosphoinositide 3-kinase delta (PI3Kδ) syndrome
Source: Front Immunol. 2026 May 7;17:1739437. doi: 10.3389/fimmu.2026.1739437 (PMC13189832; doi:10.3389/fimmu.2026.1739437)
Supplement: Supplementary file 1 [file SupplementaryFile1.docx]

Supplementary Material

# Supplementary Data

**Supplementary Material 1:** Patient narrative quotes reporting an improvement in symptoms

*“The patient reports being more energetic since beginning the study. Since COVID-19 pandemic, she has been teleworking. She walks 2 miles every day. She also goes to the gym when she is not able to get outside due to the weather.” (Patient 15, RCT).*

*“Aside from the recent joint pain issue, patient’s father reports that the patient is much improved in the year that she has been on the study drug [leniolisib], with improvements in fatigue, GI symptoms and swallowing.” (Patient 6, OLE).*

*“…she walks with no fatigue or shortness of breath. She reports her breathing is 100% better compared to last year and only uses her nebuliser twice a day.” (Patient 13, OLE).*

*“He reports increased energy and appetite, as well as improvement in respiratory status, since starting CDZ173 [leniolisib].” (Patient 4, RCT)*

*.*

*"The patient continues to do well since being started on [leniolisib], with sustained improvement in energy, appetite, and activity tolerance. He no longer has a cough and no longer produces sputum. He does not use his chest physiotherapy vest, Acapella flutter valve, or albuterol as he no longer feels that he needs these interventions.” (Patient 4, OLE).*

*"Due to the constant improvement, prednisone is tapered to 2.5 mg every other day for 1 month then stopped." (Patient 26, OLE).*

*“He continues to have a good energy level, he has walked 88 km since 11/14/16, a little more than double what he reported at his previous visit” (Patient 1, OLE).*

*“At visit 506, the patient reports that he has an increase in energy since beginning the study drug.” (Patient 11, OLE).*

*“The patient feels very well, she feels like she has sufficient energy” and, at a separate visit, “she has a feeling of having enough energy” (Patient 22, OLE).*

*“Patient has decreased incidence of colitis, gastroesophageal reflux disease and abdominal symptoms while on the investigational product at the end of the study compared to the first month.” (Patient 5, RCT).*

*“When asked what has changed in his life since he started taking the IMP, he notes, ‘my energy is higher. I had bowel movement problems where I was losing blood and that’s stopped…’” (Patient 18, OLE).*

*“She received a course of oral antibiotics for a presumed sinusitis with symptoms of cough and nasal congestion, without fever on 8/15/16 from her local doctor. She had no other respiratory or gastrointestinal symptoms.” (Patient 3, RCT).*

*“A barium swallow done just before the episode showed no significant abnormalities in swallowing pudding or solid consistencies and no aspiration or oesophageal dysmotility. Patient referred to a swallowing specialist and recommendations provided to decrease symptoms” (Patient 9, OLE)*

*“His appetite is good.” (Patient 9, OLE).*

*“Compared to the start of the study, the patient reports that his quality of life is much improved…He also reports a complete cessation of migraines, which were occurring 3–4 times per month prior to the clinical trial.” (Patient 1, OLE).*

*“I get ulcers in my mouth. They come and go. Whenever they do start coming on, I usually get three to four of them at one time. They have kind of calmed down since I started doing my study medication. They used to be worse. They don’t really hurt me.” (Patient 8, RCT and standalone qualitative interview study).*

*“The patient repeatedly noted an improvement in well-being while taking IMP…” (Patient 27, RCT).*

**Supplementary Material 2:** DFT/RCT and OLE participants (n=30) reporting symptom improvement with explicit or implied attribution to leniolisib


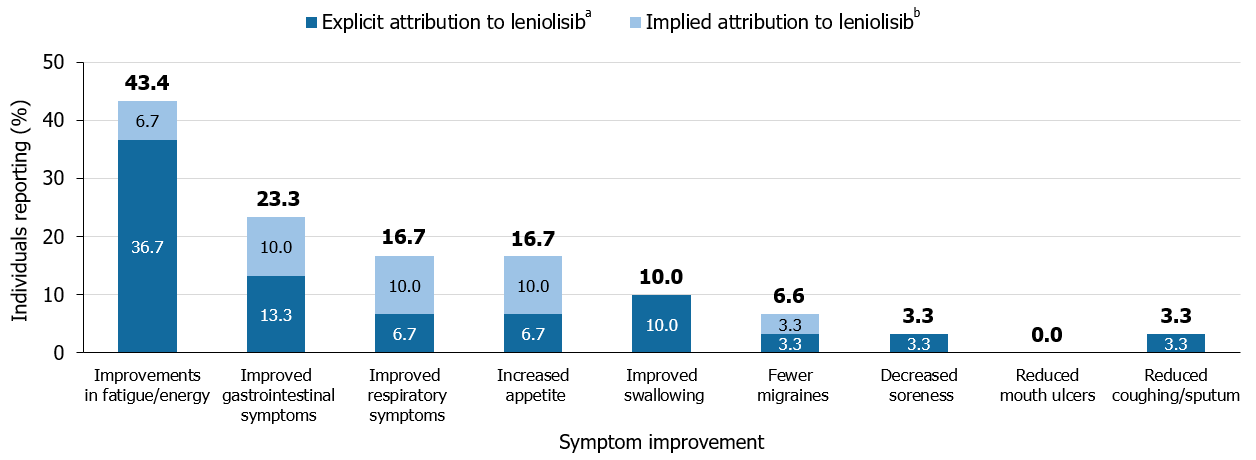


Data from clinical trials (n=30). These data only include patients’ narratives that were available from the DFT/RCT and OLE. The proportions of patients affected by each symptom at baseline were not available. ^a^Explicit attribution was defined by direct mention of leniolisib, study drug, or change from baseline. ^b^Implicit attribution was defined by a description of changes that were not specifically related to leniolisib or the time since starting treatment. **Abbreviations:** APDS: activated phosphoinositide 3-kinase delta syndrome; OLE: open label extension; RCT: randomised controlled trial.

**Supplementary Material 3:** Patient narrative quotes reporting an improvement in HRQoL impact

*“She is more active - goes cycling, goes for trips.” (Patient 21, OLE).*

*“Compared to the start of the study, the patient reports that his quality of life is much improved.” (Patient 1, OLE).*

*“Before treatment, and due to fever, weakness, and malaise, was not able to work (he is a farmer). Upon treatment [leniolisib], he was able to return fully to his profession.” (Patient 25, RCT).*

*“Due to fever, weakness, and malaise he could not perform sports or go to school. Upon treatment [leniolisib], he started going and goes normally to school.” (Patient 26, RCT).*

*“She continues to help her mom at home and recently began a part-time job. She enjoys social activities with friends and feels more energetic.” (Patient 3, OLE).*

*“He was very limited in certain activities such as travelling. Which he was able to resume upon initiation of treatment [leniolisib].” (Patient 24, RCT).*

*“Compared to the start of the study, the patient reports that his quality of life is much improved. Instead of taking 11 pills/day, he is down to 3 pills per/day. He also does not require IRT, which caused him 4 days of malaise every 2 weeks.” (Patient 1, OLE).*

*“She is happy to report that her intravenous immune globulin dose was decreased to 30 g from 35 g monthly.” (Patient 2, OLE).*

*Her mood has also improved prompting her local physician to discontinue her anti-depressant.” (Patient 13, OLE).*

*“Patient has felt better since returning to study drug. Has more energy for social activities, schoolwork and three times/week dance classes.” (Patient 6, OLE).*

*“The patient reports that he has an increase in energy since beginning [t]he study drug. He was able to travel to his home in Qatar and enjoyed social activities with friends and family while there. He is able to participate in sports activities, playing soccer and golf 3–4 times per week.” (Patient 11, OLE).*

*“As his haemoglobin improved while on study his exercise tolerance also seems to have improved.” (Patient 18, RCT).*

*“Most of my childhood was spent in a hospital bed. I never really had a childhood…life has changed for the better, thanks to a newly discovered medical condition, dedicated doctors, and access to a drug trial [OLE] with amazing results.” (Patient 4, OLE).*

*“The number of infectious episodes has decreased from 6–7 a year to 2–3 per year, which will reduce the number of working days missed due to illness. The patient repeatedly noted an improvement [in days] missed due to illness.” (Patient 27, RCT).*

*“She is enjoying her new apartment in [a new city], has made new friends and is very social with the neighbours, engaging in various games and activities.” (Patient 13, OLE).*

*“When asked what has changed in his life since he started taking the investigational medicinal product [IMP; leniolisib], he notes, ‘my energy is higher’…” (Patient 18, OLE).*

**Supplementary Material 4:** DFT/RCT and OLE participants (n=30) reporting positive HRQoL impacts with explicit or implied attribution to leniolisib


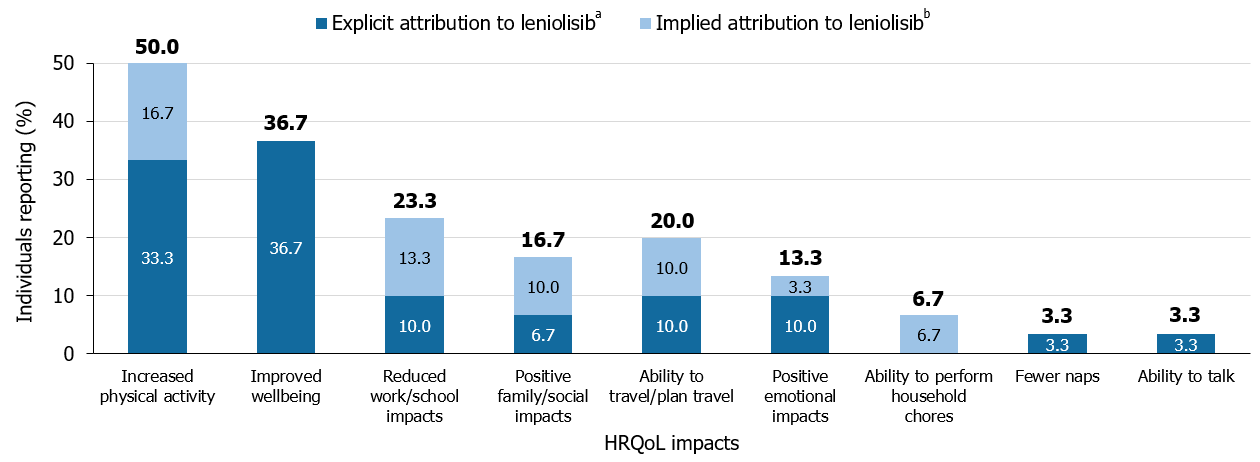


Data from clinical trials (n=30). These data only include patients’ narratives that were available from the DFT/RCT and OLE. The proportions of patients affected by each symptom at baseline were not available. ^a^Explicit attribution was defined by direct mention of leniolisib, study drug, or change from baseline. ^b^Implicit attribution was defined by a description of changes that were not specifically related to leniolisib or the time since starting treatment. **Abbreviations:** APDS: activated phosphoinositide 3-kinase delta syndrome; HRQoL: health-related quality of life; OLE: open label extension; RCT: randomised controlled trial.
