# Supplementary material for: Evaluating the impact of leniolisib treatment on symptoms and health-related quality of life in activated phosphoinositide 3-kinase delta (PI3Kδ) syndrome
Source: Front Immunol. 2026 May 7;17:1739437. doi: 10.3389/fimmu.2026.1739437 (PMC13189832; doi:10.3389/fimmu.2026.1739437)
Supplement: Supplementary file 2 [file SupplementaryFile2.docx]

**Supplementary Material 5:** Plain language summary

*What is activated PI3Kδ syndrome (APDS)?*

APDS is an ultra-rare disease in which the immune system doesn’t work correctly. People with APDS often have a wide range of symptoms that can lead to worse quality of life. These symptoms include frequent infections, cough, tiredness and chronic pain, making it hard to do regular daily activities and stay connected to others.

Leniolisib is a new medication for APDS that treats the underlying cause of the disease. A clinical trial showed that leniolisib is effective and well-tolerated when treating people with APDS aged 12 or older.

*Why was this study needed?*

In the past, standardised questionnaires, which are not APDS-specific, have been used to assess whether leniolisib changed patients’ quality of life. The results suggested some benefit of leniolisib compared with placebo. This study aimed to utilise other sources of information, including patient interviews and written records from medical professionals detailing discussions with patients to assess people’s real-world experiences and how they felt while taking leniolisib.

*What were the results?*

31 of 36 patients associated taking leniolisib with easing at least one of their symptoms or improving their lives. Half of the leniolisib-treated patients said they could do more physical activities and one third indicated that they felt healthier.

*What do these results mean?*

These results show that APDS impacts various parts of a person's life and that leniolisib may reduce symptoms and improve their lives. These beneficial effects of leniolisib add context to the clinical improvements reported in clinical trials.
